# Intervention, integration and translation in obesity research: Genetic, developmental and metaorganismal approaches

**DOI:** 10.1186/1747-5341-6-2

**Published:** 2011-01-28

**Authors:** Maureen A O'Malley, Karola Stotz

**Affiliations:** 1 Egenis, University of Exeter, Byrne House, St. Germans Rd, Exeter, EX4 4PJ, UK; 2 Department of Philosophy, Main Quadrangle A14, University of Sydney, NSW 2006, Australia

## Abstract

Obesity is the focus of multiple lines of inquiry that have -- together and separately -- produced many deep insights into the physiology of weight gain and maintenance. We examine three such streams of research and show how they are oriented to obesity intervention through multilevel integrated approaches. The first research programme is concerned with the genetics and biochemistry of fat production, and it links metabolism, physiology, endocrinology and neurochemistry. The second account of obesity is developmental and draws together epigenetic and environmental explanations that can be embedded in an evolutionary framework. The third line of research focuses on the role of gut microbes in the production of obesity, and how microbial activities interact with host genetics, development and metabolism. These interwoven explanatory strategies are driven by an orientation to intervention, both for experimental and therapeutic outcomes. We connect the integrative and intervention-oriented aspects of obesity research through a discussion of translation, broadening the concept to capture the dynamic, iterative processes of scientific practice and therapy development. This system-oriented analysis of obesity research expands the philosophical scrutiny of contemporary developments in the biosciences and biomedicine, and has the potential to enrich philosophy of science and medicine.

## Introduction

Prediction and control of biological systems are driving forces of the life sciences. While varying degrees of these capabilities have been generated in every branch of biology throughout its history, the contemporary post-genomic period has seen a remarkable surge of optimism about the possibility of achieving fine-grained control of complex interactions of biological systems. One of the many physiological systems that appears to call out for systems-based control-oriented inquiry is that of weight gain, loss and maintenance in animal bodies, particularly those of humans. Our foci in this paper are how two aspects of biological practice -- intervention and integration -- orient and configure fields of scientific inquiry and help us understand translational practice better. We suggest that these features are inseparable in ongoing research activity: to intervene successfully in complex systems requires a highly integrated, multi-level understanding that can be transferred to new contexts. Obesity research casts considerable light on this claim and illustrates the difficulties in doing so. It also offers new avenues of investigation for the philosophies of science and medicine.

Obesity science tackles the problems and causes of excess weight from a number of directions. The most well known stream of research is concerned with biochemical feedback loops and their genetic bases as well as behavioural contributions. Another rapidly developing body of research focuses on developmental and epigenetic causes of obesity and sees pregnancy and early childhood as major periods of intervention. A third, even newer avenue of research is concerned with the role microbes in the gut play in obesogenesis, and what the interventions are that might alleviate such contributions. What marks all these bodies of research is their dynamic and system-wide conception of obesity, and the integrated conceptual and methodological apparatuses that are brought to bear on the phenomenon of excess fat storage. But of equal relevance is the limited integration amongst these three research programmes, and the obstacles to their translation into effective therapies. In what follows, we outline these different perspectives on obesity, show how they overlap, and what their achievements are regarding the identification of causal relationships and the development of interventions in the process of obesogenesis. We will conclude with a discussion of the relationships between intervention-based and integrative research, and what these relationships mean for more common understandings of basic and applied research, as well as for systems-oriented strategies of inquiry. Our analysis suggests further lines of inquiry that could be pursued fruitfully by philosophers and practitioners of science and medicine, who might be able to work together on areas of common interest.

## Fat and obesity

Fat is the colloquial term for adipocytes or fat cells, which form two different types of adipose tissue: white and brown. Brown fat is a heat-producing tissue that costs the organism calories to maintain [1]. White fat is a fuel storage organ, and it is this fat that is the concern in obesity research. White fat cells are stored as adipose tissue both subcutaneously and around organs, with differential health effects [2]. Obesity is a difficult term to define. It refers to excessive and unhealthy amounts of fat (rather than body weight per se), and is measured by a variety of means, all of which have practical and theoretical problems [3,4]. But more problematic than the measuring issues are the difficulties of justifying distinctions between 'excessive' amounts of fat, mere overweight, and normal weight. Cultural factors, life history fluctuations, and health status all influence whether a particular person might be considered overweight or obese in a particular situation [5,6].

Despite such complications, obesity was declared a major health problem by the World Health Organization as early as 1985. Although excess fat storage is not a contagious disease in a literal sense, the epidemic metaphor is used to capture the rapidly rising levels of obesity and overweight in many wealthy nations [5,7]; see [8] for a problematization of this metaphor. Obesity is associated with numerous health consequences, including type 2 diabetes, hypertension, cardiovascular disease and cancers [9]. It may be both a cause and a result of these conditions. Psychological effects, such as depression, have been identified [10], and many social effects of obesity are also well catalogued [11,12]. There may even be environmental costs to obesity [13]. Recognition of these issues has made obesity into a compelling social, medical and scientific problem. As such, it cries out for effective interventions, and this need orients and drives all obesity-related research.

Even though current weight trends are necessarily correlated with 'obesogenic' environments (calorie-rich diet and low-activity lifestyle), these factors are not enough to explain obesity [14,5]. Weight-gaining predispositions (i.e., the differences between individuals) are considered by geneticists to be highly heritable, with as much as 70%-80% of weight variation attributed to genetic factors [3,15,16]. The only trait with higher heritability in humans is height. Most such highly heritable phenotypes, however, show substantial 'missing heritability'. This means that the combined effect of all identified genetic variants associated with the trait or disease can account for only a very small percentage of the difference. In the case of obesity, known genetic factors account for less than five percent of its heritability [17]. In addition, genetic causes cannot explain the massive increase of obesity from one generation to the next, nor its occurrence within a single generation as has happened recently around the world. Accordingly, scientists and public health officials recognize that there must be a complex array of factors at play in weight gain and obesity, ranging from environmental to genetic and developmental contributions, and at every level of biology in between.

## From genetics and biochemistry to physiological-behavioural systems

Throughout the twentieth century, researchers from various disciplines have conceived of weight maintenance dynamically, as the consequence of a system that connects the brain (the hypothalamus in particular), fat tissue and unknown circulating signalling molecules [18-20]. Homeostasis is the presumed default of such a system, with obesity the result of its malfunction. As obesity research embraced genetic analyses, geneticists armed with biochemical knowledge, and spurred on by epidemiological data about obesity increases, hoped initially that malfunctions in weight homeostasis could be investigated through single-gene defects [21,22]. Seldom, however, did researchers conceive of obesity as a single phenotype; nor did they think it had a single cause. Already in the early 1990s, geneticists were able to argue that human obesity genotypes will be complex multigenic systems with networks of gene-gene and gene-environment interactions. ... The growing number of obesity-related or obesity-causing genes does not bode well for the single gene hypothesis [23,24].

Early approaches to finding the specific genetic variants underpinning obesity relied on candidate-gene approaches that were based on mouse genetics and livestock breeding [25]. Candidate genes are genes that are predicted on the basis of functional knowledge to have an involvement in phenotypic traits of interest. Although there are limitations to such approaches when employed in the service of understanding complex multifactorial traits [26], such studies have nevertheless proved highly fruitful in obesity research. Through this strategy, researchers identified and characterized the first of the five known obesity-associated genes in mice, the *agouti *gene, in 1992. Agouti protein interrupts melanocortin reception in the brain (where feeding behaviour is regulated), and this results in excessive eating (hyperphagia) and obesity, as well as yellow coat colour [27,28].

Arguably an even more important finding was that of the *Lep *gene and its product leptin, from the Greek for 'thin', The recessive gene mutation (*Lep^ob ^*-- *ob *for obesity) had been linked to obesity and type 2 diabetes since the early 1950s, as was the mutant *db *of the leptin receptor gene (*Lepr^db^*) [29]. Studies of mutant *ob*/*ob *mice and *db*/*db *mice, in which the circulatory system of *ob*/*ob *mice were partly joined to lean mice, found that obesity levels in the *ob*/*ob *mice decreased. Consequently, the *ob*/*ob *mice were thought to lack circulating molecules for leanness [30]. But the *db*/*db *mice did not become leaner when their circulatory systems were conjoined with those of non-obese mice, and it was concluded they had a defect in the reception of this circulating molecule [31,32]. It took the location and cloning of the *ob *gene to work out what its product was and how it worked in relation to the receptor [33-35,29].

The functional copy of the *Lep *gene was found to encode the hormone leptin, a signalling molecule that is produced primarily in fat cells. The *Lepr *gene was identified as coding for the leptin receptor, which is located in the hypothalamic region of the brain as well as in other organs where it binds with leptin [36,37]. Despite being understood as responsible for a monogenic Mendelian disorder, the *ob *gene rapidly became known for its complicated involvement in the production of obesity. Early researchers thought that the primary function of Leptin was to make or keep organisms thin, and companies fought to win the licensing rights for leptin-based products [38,39]. Mutant leptin genes in humans result in morbidly obese individuals who are commonly glucose intolerant and insulin resistant [40]. Such leptin disorders are, however, rare. Leptin is now understood more generally as just one of the key factors in the homeostatic regulation of weight in mice and humans. When food is scarce and fat stores decrease, leptin levels are reduced, priming the organism for starvation conditions [41,42]. Once food is again available and fat stores replenished, leptin levels rise and inhibit the starvation response. Leptin appears to be informing the brain about the status of fat in the body, thereby connecting feeding behaviour, metabolism and the endocrine system to the organism's nutritional condition [43,44]. Leptin receptors connect to both feeding stimulus and feeding inhibition neurons in the melanocortin pathway of the hypothalamus. But in obese individuals, high leptin levels -- promoted by fat cells, glucose, insulin and glucocorticoid levels -- do not have the same effect on appetite and fat production due to poorly understood leptin resistance [45]. Because of this resistance, leptin replacement therapies work only on those with congenital leptin deficiences in leptin production or reception, and not on the obese with genetically standard leptin responses. The feedback loop becomes a 'vicious spiral' of ever-increasing weight gain and decreasing leptin sensitivity, especially in the context of a high-fat diet [46].

Leptin research can be seen as an exemplar of the development of an integrated molecular physiological approach. The numerous dynamic roles leptin plays in metabolism mean that its initial modelling as an anti-obesity agent has given way to a much more systemic one as an 'integrator of neuroendocrine function' [47]. The early leptin focus broadened rapidly to include a panoply of discoveries of other mechanisms and pathways, such as neuropeptides and receptors involved in short- and long-term feeding responses, as well as gastrointestinal signalling peptides [48,49]. In these interlinked systems, leptin plays a major but not the only role in appetite control and metabolic function.

Increasing knowledge about leptin has also led to the reconceptualization of the role of white fat cells from inert storage devices to highly active endocrine organs [2]. The secretions of these cells (including about 50 other proteins, collectively called 'adipokines', plus other secreted substances) communicate throughout the whole body. They are receptors that interact with a vast range of compounds and are involved in almost every bodily process from reproduction to metabolism and immunological response [50]. Adipokines affect glucose levels, appetite and the function of many organs [51]. In obese individuals, adipose tissue is the largest and most highly variable endocrine organ in the body, and its very distribution pattern (android or gynoid) has major health implications. There is considerable evidence that adipocytes are dysfunctional in obese organisms, and many obesity researchers are convinced that progress will depend on understanding adipose tissue as a developing, regulatory and metabolizing endocrine system [52]. Brown adipose tissue, long thought irrelevant to mature human physiology, is now known from a broader system perspective to have a role in how adipose tissue is maintained in obesity [53]. White adipose tissue is composed of more than adipocytes, and also includes macrophages, endothelial cells and fibroblasts [54]. Some of these cells (possibly adipocytes themselves) produce insulin-desensitizing inflammatory molecules, and a number of researchers now believe that obesity is a low-grade inflammatory disease that causes diabetes [55,50,56]. But little so far is known about pathways and mechanisms for adipose tissue-derived inflammation and its relationships to insulin and leptin resistance. Further exploration of this connection seems bound to make the obesity story yet more complex.

As part of the attempt to generate a system-wide account of obesity, genome-wide linkage and genome-wide association studies search not for biologically implicated candidate genes, but for particular markers across the genome that are linked to disease [57,15]. This shift to a bioinformatically driven analysis, often thought of as discovery-driven, has produced numerous associations of genes or genetic regions with obesity, especially in groups with high rates of obesity or in less obesogenic environments [58]. Subsequent efforts to identify mechanisms and biochemical pathways between genes and obesity phenotypes have, however, been slow and halting [59]. The *FTO *gene is the most robust finding of genome-wide approaches, but it is associated with only a few kilograms of weight gain, and studies of its functional role have only just begun [60,61].

The quest for a full understanding of obesity will not be satisfied by genetic and biochemical explanations, however, even if these are inevitably woven into neurochemical and behavioural explanatory accounts [62]. This is particularly the case because of the limited effectiveness of pharmacological interventions in obese adults and because of the alarming rise in rates of childhood obesity [63]. Some of the genes implicated in obesity phenotypes, such as the *FTO *gene, may also play a role in epigenetic processes that through effects on satiety regulation can result in increased fat stores [64]. This broader dimension, of epigenetic and developmental analyses of the differential expression of genes, is increasingly important in the investigation of fat production across the lifetimes of individuals and families.

## From evolutionary to developmental and epigenetic aspects of obesity

Genetic approaches to obesity have been instrumental to early evolutionary explanations for the rise in obesity level in Western societies. The first such theory was the 'thrifty gene hypothesis', which was formulated in the sixties by geneticist James Neel [65]. According to Neel, thrifty genes were favored by natural selection because they conferred protection against the regular famines our hunter-gatherer ancestors experienced. In contemporary society, however, such genes predispose individuals to obesity and diabetes [65] (see [66] for a critique). John Speakman's 'predation release hypothesis' [67], in contrast, argues that early human adaptations for slimness have been recently displaced by a decrease in selection pressure, which has allowed relevant genes to drift towards obesogenic predispositions. Competing against such gene-based evolutionary accounts were those claiming environmental causes, such as high-fat diet and sedentary lifestyle, are responsible for the immense increase in obesity in affluent societies. This perspective was radically retheorized in the early 1980s by clinical epidemiologist David Barker. He detected a positive correlation between poor conditions in childhood, which he measured with infant mortality rates and birth weight, and later metabolic diseases [68]. His studies showed that early prenatal exposure to adverse environmental effects had immediate effects on birth weight, which created predispositions to late-onset lifestyle diseases such as obesity, type 2 diabetes, hypertension and cardiovascular disease [69,70]. From these findings Barker formulated his 'thrifty phenotype hypothesis', according to which the fetus reacts to circumstances in its environment through vascular, metabolic and endocrine adaptations [71].

A flood of epidemiological and laboratory-based animal studies has strongly supported Barker's original claims. Some of this experimentation has shown that different disease phenotypes can be produced by differential gene expression [72-75]. This perspective has been labelled more broadly 'the developmental origins of health and disease' (DOHaD) paradigm [76]. Paediatrician and endocrinologist Peter Gluckman and colleagues have argued that the thrifty phenotype hypothesis and DOHaD should be understood as a subset of the more encompassing processes of 'developmental plasticity' that allow organisms to adapt to a suite of different environments with the most suitable phenotypic variant. The placenta is thought to play the key role in what is often referred to as 'fetal programming', which involves both structural changes to major organs and the epigenetic modification of key genetic factors [77]. Some researchers reject the biologically problematic metaphor of 'programming' because of its connotations of genetic preformation [78], but in this field it has been adopted to communicate that the fetus treats the nutrient supply through the placenta as a forecast for the availability of food after birth, and prepares for this with a modified developmental trajectory [72]. The organism adapts itself teleologically (with the goal of survival) to its environment, and this adaptability -- produced by phenotypic plasticity -- is an evolved capacity of such organisms.

Gluckman has explained the long-term effects of the maternal environment on offspring not simply as responses to developmental disruption, nor merely as short-term adaptive responses. He understands them rather as 'predictive adaptive responses' (PARs). Early environmental cues shift the developmental pathways in order to match the phenotype to the projected environment [75]. These PARs manifest their adaptive effects later in life rather than immediately. The advantage of such a plastic strategy depends crucially on the accuracy of the forecast of the postnatal environment. A thrifty phenotype with a high ratio of fat to muscle cells, a highly efficient metabolism, weight-gaining appetite and exercise regulation may have clear advantages in an environment with poor nutritional supply. In an environment with an overabundance of high-fat food, however, such a phenotype is likely to lead to high weight gain and an increased risk of associated diseases. Gluckman and colleagues name this scenario the 'environmental mismatch hypothesis'. They thus embed their developmental explanation within a broader evolutionary analysis [79].

While epidemiological studies have been very important for detecting strong associations between certain phenotypes and distinct developmental factors, they are only guides to the causal bases and potential points of intervention in syndromes such as obesity. Only in the last few years have molecular bridges been made between periconceptual (prior to conception), prenatal and perinatal (around the time of birth) environments, long-term changes in gene expression of normal housekeeping genes, and permanent changes in adult morphology, physiology and behavior [71]. The observed plasticity in human and non-human developmental trajectories is believed to be achieved largely through the altered expression of key regulatory genes that regulate cell number and differentiation early in development, thus permanently resetting many homeostatic mechanisms [71]. These epigenetic factors can play a major role in weight gain in later life.

The term 'epigenetics' was originally coined by Conrad Waddington as the synthesis of 'genetics' and 'epigenesis' in reference to the interaction between genetic, cytoplasmic and environmental factors as they construct the phenotype [80]. In today's molecular biology, epigenetic modifications refer to heritable changes in phenotype that are not caused by changes in the underlying gene sequence but arise in dependence on the molecular modification of the DNA or posttranslational modifications of the proteins that package the DNA. These mechanisms programme the time- and tissue-dependent expression of genes and the differentiation of cells from their totipotent state as stem cells to their final phenotype as differentiated somatic cells of various tissue types. Experimental evidence in epigenetics has associated specific disease risks with environmental exposure during narrow time-frames. An increased risk for obesity has been clearly linked to maternal malnutrition in the first trimester [81,74]. In cloned animals, nutritionally challenged maternal environments as early as the periconceptual and pre-implantation period can lead to long-term effects on metabolic function [82,83]. In humans, in vitro fertilization confers a higher risk of obesity later in life, possibly due to low methylation (hypomethylation) in culture conditions of typically methylated and therefore silenced maternal alleles such as the insulin-like growth factor II (*IGF-2*) [84].

The most well known evidence for epigenetic changes in humans induced by maternal malnutrition comes from the natural experiment created by the famine of the Dutch Hunger Winter of 1944-1945. A study of subjects exposed to famine conditions, particularly during periconceptional and early fetal periods, revealed an association with the hypomethylation of the *IGF-2 *differentially methylated region six decades later [80]. Because the hypomethylated gene is expressed, these subjects *and *their children showed a significantly increased incidence of obesity and diabetes. However, this hypomethylation was not associated with lower birth weight, which is instead associated with exposure to famine in a later prenatal period. This study reinforces the supposition that very early development is a crucial period for establishing and maintaining epigenetic marks [81].

But these accounts are dependent on maternal malnutrition, which is not the case for most mothers in Western societies, with the notable exceptions of less affluent social groups or women following extremely restricted diets. Experimental evidence points to several developmentally plastic processes that increase the risk of developing obesity in an environment with easy access to high-energy food. One pathway leads from prenatal malnutrition and -- importantly -- high glucocorticoid exposure due to stress, to an increased sensitivity to an obesogenic environment. Such malnutrition of the fetus can also be induced by a restricted placental supply associated with early, late and first pregnancies. A second pathway predicts a higher risk for obesity later in life following maternal high-fat and low protein diet, obesity and diabetes. These antenatal challenges can be postnatally amplified through infant overfeeding, which includes the so-called catch-up growth often experienced by low-birth weight children [71]. The most impressive experimental evidence for prenatal changes in epigenetic gene expression as a response to maternal cues involves the glucocorticoid receptor (GR) and the hypothalamic-pituitary-adrenal axis related to the stress response. The GR binds glucocorticoid, a hormone involved in the stress response pathway, which has multiple behavioral and metabolic functions, including inflammation and insulin interference. Hypermethylation and underexpression of GR in the rat brain and liver results from an excessive exposure to maternal glucocorticoids in the womb, as well as from prenatal protein restriction and changes in maternal care behavior immediately after birth [85,86].

Another early candidate gene for the modification of epigenetic expression due to maternal nutrition, and in preparation for a nutritionally challenged environment, is the *IGF2 *gene [87]. Under normal conditions it is maternally imprinted and hence only expressed from the paternally inherited allele. A great range of other genes have been implicated in epigenetic alteration after exposure to either malnutrition, maternal stress, or an oversupply of glucose and fat. Many of the long-term effects experienced by the first-generation offspring have been inherited by the second, and occasionally even third-generation offspring [88]. This is not surprising considering that in females the primary oocytes (egg cells) develop well before birth, thereby gaining exposure to maternal cues for the next generation. In general, epigenetic mechanisms can cause environmental cues experienced by the parent to shift the offspring's developmental pathways, which then modify the phenotype in 'expectation' of a later environment. This process is sometimes described as 'spite the mother -- fight the offspring' [89].

The potential for finding epigenetic causes of obesity and other metabolic diseases promises increased chances of medical and public-health intervention. The PAR paradigm has shown that the more molecular insight is gained into the influence of environmental cues on the phenotype, the more sophisticated an understanding of gene-environment interactions is needed. Environments matter in several ways: as immediate versus long-term effects on gene expression, and as past gene-environment interactions that strongly influence present gene-environment interactions. Consequently, the complex interaction of their combined influences on a phenotype is what dictates the path for intervention. Two interdependent approaches to reduce the risk of obesity and related diseases later in life can be extracted from the PAR paradigm. First, promoting the health and nutrition of female reproducers may prevent chronic disease in future generations [75]. Second, intervention can attempt to manage the postnatal infant in accordance with its prenatal programming. There may also be opportunities for drug-based intervention. It has been shown in animals that leptin administration in the sensitive period after birth can partly reverse the course of development towards obesity [73,90]. What connects these two approaches to intervention is the biology of epigenetics.

Intervention is enabled by extensive knowledge of the enzymes involved in the establishment, maintenance, and removal of epigenetic marks both of the DNA and its histone proteins, and of the mechanisms that link environmental cues such as behavior or diet to epigenetic mechanisms [91,84]. Generally, exogenic influences such as behavioral or nutritional exposure trigger specific signaling pathways, which in turn activate sequence specific elements such as transcription factors, enhancers, or miRNAs. Knowledge of these pathways and their adaptive origins may open up possibilities for both the prevention of gene expression pathways in the fetus and the correction of already programmed, developmental responses to early cues. These sorts of interventions would decrease susceptibility to obesity after birth [92,93]. None of this will be simply achieved, but it can be supported by a broad public-health orientation to intervention that is generally focused on maternal well-being.

## From single-organism to metaorganismal approaches

It may have seemed as if epigenetic and developmental explanations of obesity came out of nowhere in the mid-1990s and contributed a rich vein of insight to obesity studies. But at the end of the 1990s, from yet another direction, an altogether different account of obesity emerged. Under the banner of microbiology, bringing together microbial ecology and genomics, came the study of gut microorganisms and their functional effects on human biology. The prevalence of bacteria such as *Escherichia coli *in the human gut has been known since these organisms were isolated and named after Theodor Escherich in 1885. But only from the 1970s onwards has the contribution of these microorganisms to human physiology and development been a real focus of attention, beginning with Dwayne Savage's [94] estimates of the 10:1 ratio of microbial and human cells in the human body. Molecular studies throughout the 80s and 90s determined important contributions from microbes to human metabolism, the immune system and development [95,96], but it took the genomic era to bring home the true significance of microbial participation in human health.

The sequencing of single microbial genomes was established well before animal genome sequencing, but the advent of metagenomics -- the sequencing of entire microbial communities in their natural environments [97] -- made it clear that the exclusively human genome sequence needed supplementing by the sequence of the human microbiome. The microbiome is the collective genome of the microbial community dwelling in and on an organism, and knowledge of the entire community's metagenomic sequence (outnumbering human genes at least 100:1) is considered crucial for a full understanding at a molecular level of the relationships between human hosts and their microbial constituents [98,99]. The largest microbial community in the human body is found in the gut, and much human microbiomics has focused there. One of the important early findings of these community-wide molecular studies of human gut microbes was the difference between the gut microbiomes of obese and non-obese hosts. In fact, obesity appears to have major implications for the composition of human gut microbiomes, and vice-versa.

The gut microbiome has co-evolved with humans and taken on some of the metabolic function necessary to human survival, such as vitamin biosynthesis and plant polysaccharide (starch and cellulose) fermentation [100,101]. Gut 'microflora' play crucial roles in the developing human organism, and contribute to immunological processes (e.g., inflammatory response), endocrinological functions, and homeostatic energy balancing such as energy extraction and fat storage [102,103]. 'Germfree' mice, raised from birth in sterile conditions, are significantly thinner than microbially colonized mice, despite eating the same amounts of food [104]. When experimentally colonized by normal mouse microbiota, these previously germ-free mice become fatter and attain much higher levels of leptin production and insulin resistance [105]. This implies that the caloric value of food is relative to the microbiotic ability to extract energy from otherwise undigested material [106]. In a feedback loop, diet is 'imprinted' on metabolism through its selective effects on the microbiota [107].

In obese mice and humans, the proportion of Firmicutes (a large and diverse phylum of bacteria) to Bacteroidetes (a smaller and less diverse phylum) is greater than in lean organisms. When these same obese individuals lose weight, the ratio alters, and becomes closer to that found in lean organisms [108,104] (but cf. [109]). Obese (*ob/ob*) mice, with 50% more Firmicute activity in their guts, appear to digest more of their food and gain more calories from it, and the Firmicutes also regulate host metabolic genes [110,111]. A more detailed investigation of the composition of mammalian gut microbiota is ongoing, including scrutiny of the interactions between hydrogen-producing archaea and hydrogen-consuming bacteria [112,104]. While there is considerable microbiotic variation between human individuals, a core microbiome of metabolic functions is maintained regardless of the diversity of taxa represented [113,97], thus implying considerable functional redundancy in the microbiome. These core functions intersect in very important ways with host genetics and development.

Humans and other animals experience a developmental succession of the composition of their gut microbiota, driven partly by dietary changes and partly by differential exposure to microbes, as well as generally by host genetics [114,115]. Initial colonization of infants is highly variable -- even 'chaotic' -- but later interactions between the main bacterial groups, as they attain a composition more similar to adults, reaches an equilibrium that brings about standard developmental effects in gut formation [116]. Microbes link human breast feeding and weight gain in poorly understood ways. Children with developmentally unusual gut microbiota appear to have predispositions to obesity, gut inflammation and higher allergy rates [117]. Gut microbes are also implicated in the development of type 1 diabetes, through epigenetic effects on the innate immune system [118]. Humans who have undergone gastric bypass surgery as obesity therapy have a microbiomic composition that is different from both obese and slim individuals [119].

One potential mechanism for such microbially mediated differences in weight gain is the microbial suppression of Fiaf (fasting induced adipocyte factor) in the gut. This suppression leads to increased deposits of triglycerides in fat cells [109]. Not only are the microorganisms increasing the energy harvest in the gut, but they are also affecting the regulation of how this energy is stored and how the immune system operates [120,121]. The latter is important because imbalances in the composition of the microbial gut community can lead to inflammatory diseases, and such inflammation can be linked to obesity [122,123]. But the exact relationships between obesity, leptin, diet, inflammation and microbiotic variation are still obscure, and a great deal more work on humans as well as gnotobiotic model organisms (germ-free animals exposed to specific microbes) has to be done to work out the mechanisms and regulatory factors that contribute to microbially influenced obesogenesis, associated syndromes and their maintenance. Nor is much known about the effects of large numbers of viruses in the human gut (also detected in metagenomic surveys) and whether they modulate microbial activity.

Currently, little epidemiological research has been done on microbial community composition and disease in large populations, although metagenomic techniques enable such associations to be drawn. One current study, drawing on the 'thrifty' metaphors noted above, is called the 'thrifty microbiome' project. It seeks to understand the role of gut microbiota in producing obese phenotypes in a genetically similar and well characterized population, the Amish [124]. It is recognized that metagenomic approaches on their own will not be enough to reveal these host-microbe interactions, and that they will need iterative extension by experimental techniques and multiple modes of analysis [119,112].

Although the interactions between microbial, genetic and epigenetic processes in the generation of obesity have yet to be illuminated, the identification of a whole new dimension of obesity factors may enable the development of novel diagnostic, preventive and therapeutic interventions (Bäckhed et al. 2004). A great deal of nutritional research is focused on prebiotics (non-digestible food used to stimulate existing gut microbes) and probiotics (live microorganisms as a dietary supplement), but the diversity of products and non-standardized study designs make it difficult to compare the efficacy of such interventions [125]. Claims that probiotics are causally linked to obesity are deeply disputed [126]. Perhaps more significantly, antibiotic ingestion affects the composition of gut microbial communities and is linked to weight increase in cattle and other animals, especially if they are exposed to antiobiotics early in the lifecourse [127]. The treatment of young children with antibiotics may be playing a role in the obesity epidemic. And drug therapies for any disease are regulated by microbial interactions, so any attempt to predict pharmacological efficacy has to take gut microbes into account [128].

Metaorganismal studies of obesity are -- of the three research approaches discussed in this paper -- perhaps the most 'curiosity'-driven rather than intervention-driven, in part because of the novelty of the approach and considerable associated uncertainty. Some gastroenterologists urge caution in applying metagenomic findings to public health policy because so little is known and obesity effects are obviously driven by a panoply of causal factors [124]. Nevertheless, such studies are broadening understandings of physiological processes rather than narrowing them, and this is happening at the conceptual level as well as the level of potential interventions.

It is becoming clear to many researchers of obesity that microbes demand a broader understanding of what an organism is, and how its interactions with the environment are mediated [129,100,127]. It is common now to theorize the trillions of microbes in the human gut in each and every human as a metabolic organ in its own right [130]. Metaorganismal approaches also emphasize different modes of inheritance. Obesity propensities are not restricted to genetic and epigenetic inheritances, but are acquired laterally, from the mother and wider community. This lateral mode of inheritance becomes interwoven, of course, with the epigenetic and genetic modes and the physiological story becomes even more complex and dynamic. Further complications are introduced with the evolutionary aspects of obesogenic factors, such as the complex adaptive and selective processes that structure microbial communities in the gut [131,132]. What does seem clear, however, is that increasingly sophisticated understandings of the factors involved proceed hand-in-hand with a search for increasingly sophisticated interventions, and that in the process, a new, far more dynamic understanding of basic and applied categorizations of scientific research is beginning to emerge.

## Intervention, integration and translation

These three different approaches to obesity each tell a partial and poorly connected story of obesogenic mechanisms. The first approach looks for genetic causes, employing genome-wide association studies, whole-genome linkage scans and molecular techniques to identify the molecular pathways involved. The second approach uses epidemiological studies, animal experiments and molecular approaches to address gene-environment interactions that manifest themselves epigenetically early in life. The third line of inquiry investigates variations in the composition of gut organisms and their relationship to obesity via epidemiological association studies, metagenomic techniques and biochemical analyses. All these approaches may have hoped initially for linear, one-dimensional and unicausal explanations but these were obviously ill-founded hopes. Assuming simple linear pathways as the causal explanation of disease has produced neither promising preventive strategies nor effective drug treatments for obesity, as the leptin story shows. While there may be diseases and biological processes for which multi-level systems explanations are unnecessary, we focus on obesity as the exemplar of a disease for which a narrow causal story and limited range of interventions will not suffice. Obesity, along with many other common conditions, has to be addressed at multiple levels, including the social [133,134], and ultimately all three of the approaches outlined in this paper have had to widen their focus and begin to construct much more multifactorial cause-and-effect scenarios. Philosophers of science and medicine have long argued for the necessity of multilevel explanation (e.g., [135-137], and the era of systems biology and systems medicine is seeing the gradual implementation of complex causal accounts of disease.

Even though each angle of inquiry into obesogenesis has generated a range of more sophisticated understandings, the connections between these three major perspectives have barely been investigated. We have pointed to a few links made between, for example, the genetics and epigenetics of obesity, or developmental and microbiological interactions, but this is just the beginning of developing a genuinely organism-wide and dynamic life-history understanding of obesity. Such connections need to be made not simply for theoretical satisfaction, but for the practical benefits that arise out of greater capacities for the prediction, prevention and management of obesity. Such aims are part of the translational agenda of much biomedical research today, and we suggest that this agenda rests squarely on the shoulders of research capacities for intervention and integration. We suggest that what will bring these three programmes of obesity research together is a systems-oriented translational undertaking that frames the future of obesity research as it might be approached by systems medicine.

***Intervention ***is the ground on which explanations of biological processes are built. Interventionist science involves the search for causal-mechanical explanations of phenomena [138]. Causality, from an interventionist perspective, cannot be inferred merely from observation of a biological process but requires experimental manipulations that take control of one or more variables [139]. Conceiving of intervention as the core of scientific practice provides a useful way in which to comprehend the life sciences in general, and obesity research in particular. First, an intervention that is successful in one research setting has to be transferred successfully to others in order to be maintained as a robust causal inference. And second, understanding translation as the successful transfer of that intervention aids the development of a broader understanding of translation, in which knowledge is understood as a practical accomplishment. We are not denying that sophisticated understandings of biological processes can be built on initially restricted combinations of techniques, questions and bodies of data. Very specific questions may continue to be fruitfully addressed by particular techniques and specific mechanistic accounts. However, the recent history of molecular biology, and obesity research in particular, shows that even as limited approaches succeed, they generate a requirement for the integration of more data and approaches in order to answer associated or wider-ranging questions [140].

This ramification of inquiry is what each of our three accounts of obesity research demonstrates. For example, an experimental manipulation of the leptin-producing pathway, so that it suppresses the glucose production stimulant, glucogen, and leads to the normalization of glycemia in insulin-deficient rodents, suggests a potent therapeutic intervention point [141,142]. But this experimental intervention would need to be reproduced in a variety of experimental systems (e.g., different animal models, different conditions of calorie intake) and integrated into a wider body of intervention-oriented knowledge (e.g., insulin regulation). If such an intervention proves robust, despite its artificial isolation from a wider system of processes, it may be transferrable to a context of therapy development. However, as our outline of the history of leptin research made clear, focusing on a single point of intervention is not likely to bring about an effective and general therapeutic strategy for a broad condition. As bodies of molecular physiological knowledge have been generated, and multilevel insights into complex processes such as obesity have expanded, *integration *has become the catchcry of a forward-looking research agenda. This agenda is aimed at the greater transferability of research findings from one research domain to another, and the subsequent expansion of prediction and control this may enable.

***Integration ***has emerged as a major desideratum in an era of biological practice that is characterized by the high-throughput production of vast bodies of data and extensive powers of computational analysis. An increasingly prominent response to this embarrassment of riches is systems biology, which mandates integration at all levels of practice: methods, materials, data, causal inferences and disciplinary approaches [143-145]. This new approach is characterised by the effective combination of experimental 'wet' biology with computer-based analyses and mathematical models [144]. Through the repeated integration of biological knowledge, predictions made from mathematical models can be tested experimentally, the models modified accordingly, more data integrated, and at each step, systems-level insight improved [146].

Integration cannot stop in the laboratory, however, and one of the justifications of systems biology is its mooted ability to connect more closely and immediately with 'translated' benefits. The 'systems medicine' manifesto is a prime example of such an integration, where efforts are made throughout the whole research process to integrate systems-biological insights with clinically applicable results [147-149]. From this perspective, conditions such as obesity have to be understood as the consequences of complex interactions between networks of molecular activity and environmental factors. These understandings are deemed to require iterative research strategies taking multiple levels of data into account [150-152]. The main goal for these systems-oriented practitioners is not exclusively understanding or explanation but the prediction and control of systems so that basic and applied scientific practices are not separated. 'Knowledge' in such a research context means being able to intervene in a particular process and affect its outcome in a variety of contexts. Even where a systems approach does not immediately lead to increased control, it produces a better understanding of the system.

It might be thought that intervention, with its focus on linear cause-effect relationships, is at odds with system-level integration. For a therapeutic intervention to be effective from a systems point of view, it should be based on multilevel insights that pervade the whole organism, its life-course and environment [153,139]. Yet, any experimental or therapeutic intervention, no matter how broadly informed and carefully conducted, is going to operate as a simplified modification of self-maintaining multidimensional biological processes. But just as experimental interventions focus on the manipulation of particular variables in order to understand causal relationships, systems-biological manipulations focus on nodal points of intervention from which wider system effects can be predicted [154,151]. Manipulation of these nodes is achieved on the basis of knowledge about the interconnections between and within systems. Identifying the multilevel effects of nodal interventions allows better prediction and control not just of that node but those connected to it. Nodal interventions must also take steps to predict and avoid side-effects and compensations, which are serious problems for single-pathway pharmaceuticals that attempt coarse interventions in dynamic systems [155]. The aim, therefore, of systems biology and systems medicine is to increase the effectiveness of experimental and therapeutic interventions, rather than simply extend them to multiple levels and aspects of the system. By framing complex processes pragmatically, translation brings together integration and intervention and potentially resolves some of the apparent contradictions between them.

***Translation ***is often discussed as a key issue for contemporary biosciences. It is usually conceptualized as the transformation of knowledge into useful products [156-158]. In medicine and clinical practice, where translation is a frequently discussed process, it is described as the strategies that move research 'from bench to bedside' [159,160]. Although there is considerable dispute about how to conceptualize and implement translation, we suggest it cannot be restricted to the linear transition of research from lab to clinic or industry, because of the impoverished account of scientific practice on which this definition relies. Rather than discussing translation in this restricted sense, we urge a broader understanding of translation as the transferrable achievement of intervention. Going back to obesity research for exemplification of this point, it is scientifically desirable that a laboratory-based biochemical intervention in, for example, leptin and insulin interactions in mice, is potentially transferrable to other experimental systems and other disciplinary approaches. And ultimately, genetic and biochemical understandings of leptin and insulin interactions have to be integrated into epigenetic and metaorganismal knowledge, or the successful transfer of intervention will fail. The rationale here is not purely theoretical or philosophical (i.e., whole systems are the best objects of inquiry) but pragmatic: concerned with the transfer of a particular intervention into another context.

Systems biology is not, in this scenario, a replacement paradigm for the reductive investigative strategies of the molecular sciences but their necessary extension in certain inquiries. As we have noted, some conditions and their associated explanations and interventions may not require full systems-biological and systems-medicine approaches. Systems biology and medicine seek to integrate not only approaches and data, but also discovery, analysis and therapy in a variety of contexts. We suggest that this confluence is where the true meaning of translation lies: in the capacity to transfer interventions from context to context during the pluralistic investigation of a system. These contexts are simultaneously rather than sequentially producers of experimental as well as application-oriented interventions. A therapeutic intervention, such as leptin replacement, will not achieve much in organisms that are not leptin-deficient but leptin-resistant. But this very failure of therapy can be conceived as a process manipulation and transferred into further experimental contexts to enrich understanding of success and failure of that intervention. Translation also encompasses the integration of what might be thought of as more philosophical perspectives on scientific practice. The obesity research narratives we have told show how initially fruitful reductionist strategies are being translated into the investigation of nonlinear interactions between components in multidimensional systems. Such system-oriented insights are restricted at first, but can eventually be transformed into more dynamic, interactive representations of the system under study, as each of our three bodies of research shows. In this framing of contemporary biological practice, obesity research is a prototypical example of the requirement to understand the dynamic of science in translational terms. By 'requirement' we mean not simply something mandated from above (e.g., by funders, health policy, and profit incentives) but as a descriptive and normative account that encompasses scientific practice in today's biology.

Thus, from the perspective we advocate -- which is highly compatible with several philosophical positions on pluralism, pragmatism and intervention -- translational activities occur at every level of scientific practice, such as when predictions are translated from one disciplinary or explanatory domain to another through transferable technologies and generalizable model-based insights. These cumulative processes of translation are always concerned with intervention, whether in experimental systems, clinical trials or product development. Thinking in this way about translation clearly challenges the distinction between basic and applied research -- already argued by many commentators to be a false categorization [161-163]. A broad conception of translation also includes fundamental theoretical changes and the exploration of new avenues of research occurring in the same contexts in which new interventions are being developed and applications anticipated. Such interventions thereby become part of the iterative cycle of translation, further intervention and reintegration.

Very clearly, therefore, obesity research is a field that can contribute to deep insight in the philosophy of science and medicine. As our outline of the three streams of research shows, philosophical accounts of causality, intervention, pluralism and explanation are given new and pressing reasons to be used as tools of analysis in regard to biomedical research programmes. Philosophers will gain valuable understanding of systems perspectives on health and illness, as well as the process of translating scientific knowledge and tools from one context to another within and between areas of research and application. These philosophical insights have the potential to contribute different levels of insight to emerging areas of bioscientific and biomedical research even as philosophy develops novel concepts and frameworks to accommodate this new knowledge. By thinking of the relationship between science and philosophy of science as interactive and mutually informing (e.g., [164]), greater joint capacity can be developed to address research impact, critical limitations, and new modes of scientific practice. It should not be necessary to argue (although sometimes the need is felt e.g., [165]) that an informed philosophical analysis of the science is also required for better ethical analysis. We suggest, therefore, that understanding the epistemic and ethical complexities of obesity research will illuminate the many practical scientific issues arising from the development of therapeutic interventions and the policies they are likely to inspire. This illumination will have consequences for other fields of medical and scientific research, thus resulting in further mutually beneficial interplay between science, medicine and their philosophical studies. Obesity, in all its dimensions, is able to expand philosophical discussion while the philosophy of science adds new angles of discussion to the study of obesity.

## Competing interests

The authors declare that they have no competing interests.

## Authors' contributions

MAO and KS contributed equally to the research, analysis and writing of this paper. Both authors have read and approved the final version.

## Author information

Both of the authors are philosophers of biology, with general interests in the history and philosophy of science. They have each made close engagement with scientific fields a feature of their research. MAO is a senior research fellow at Egenis, the University of Exeter. She will move to the University of Sydney in February 2011. Her work is broadly concerned with the history and philosophy of microbiology, and more specifically, with systems and synthetic biology. KS, an Australian Research Fellow at the University of Sydney, is an internationally acknowledged expert in the history and philosophy of molecular biology and epigenetics. Both authors have published extensively in humanities and scientific journals.

http://socialsciences.exeter.ac.uk/sociology/staff/omalley/, http://paul.representinggenes.org/Stotz/
